# Effectiveness of a digitally supported care management programme to reduce unmet needs of family caregivers of people with dementia: study protocol for a cluster randomised controlled trial (GAIN)

**DOI:** 10.1186/s13063-021-05290-w

**Published:** 2021-06-16

**Authors:** Olga A. Klein, Melanie Boekholt, Dilshad Afrin, Christina Dornquast, Adina Dreier-Wolfgramm, Armin Keller, Bernhard Michalowsky, Ina Zwingmann, Stefan Teipel, Jochen René Thyrian, Ingo Kilimann, Wolfgang Hoffmann

**Affiliations:** 1 Deutsches Zentrum für Neurodegenerative Erkrankungen (DZNE), site Rostock/Greifswald, Rostock, Germany; 2 Deutsches Zentrum für Neurodegenerative Erkrankungen (DZNE), site Rostock/Greifswald, Greifswald, Germany; 3 Institute of Medical Psychology and Medical Sociology, Medical Faculty, University of Rostock, Rostock, Germany; 4 Department of Nursing and Management, Faculty of Business and Social Sciences, Hamburg University of Applied Sciences (HAW), Hamburg, Germany; 5 Department for Psychosomatic and Psychotherapeutical Medicine, University Hospital Rostock, Rostock, Germany; 6 European University of Applied Sciences (EU FH), Rostock, Germany; 7 Institute for Community Medicine, Section Epidemiology and Community Health, University Medicine Greifswald, Greifswald, Germany

**Keywords:** Caregiver of people with dementia, Caregiver health, Unmet needs, Care management system, Cluster randomised controlled trial

## Abstract

**Background:**

Up to two-thirds of dementia care is provided by family caregivers who often experience high burden, little support and adverse health outcomes. Enabling and supporting family caregivers to provide care at home prevents early institutionalisation of the person with dementia and alleviates the economic burden of dementia in the long term. General practitioners (GPs), as the first point of contact, have a key role in identifying and managing burden and care needs of family caregivers. However, in routine care, this opportunity is often limited by time constraints and even if caregiver needs are recognised, detailed information about regionally available support and advice on healthcare services is often lacking.

**Methods:**

This is a cluster randomised, controlled trial investigating the clinical use and cost-effectiveness of a digitally supported care management programme for caregivers of people with dementia (PwD). Five hundred family caregivers will be randomised at GP offices, specialist practices and memory clinics, with about *n*=250 participants per arm. Participants are eligible if they are the primary family caregiver of a PwD, are at least 18 years of age and provide informed consent. Participants in the intervention group will receive an individualised care management plan, which will be carried out by qualified study nurses in collaboration with the treating GP. All participants will receive a baseline assessment and a 6-months follow-up assessment. Participants in the wait-list control group will receive usual care. Starting at the 6 months’ follow-up, the former controls will also receive an individualised management plan. Primary outcomes are the number of unmet needs (incl. the Camberwell Assessment of Need for the Elderly, CANE) and health-related quality of life (EQ-5D-5L) at 6 months. Secondary outcomes include caregiver burden (Zarit Burden Interview, ZBI), social support (Lubben Social Network Scale, LSNS), the use of medical and non-medical services (Questionnaire for the Use of Medical and Non-Medical Services, FIMA) and resource utilisation (Resource Utilisation in Dementia, RUD). The primary analysis will be based on intention-to-treat. Between- and within-group analyses and a cost-effectiveness analysis will be conducted to estimate the effect of the tablet PC-based care management programme. This trial is funded by the German Federal Joint Committee (G-BA) Innovation Fund.

**Discussion:**

The findings of this trial will be useful in informing and improving current healthcare system structures and processes to support family dementia caregivers within routine care practices.

**Trial registration:**

ClinicalTrials.gov NCT04037501. Registered on 30 July 2019.

**Supplementary Information:**

The online version contains supplementary material available at 10.1186/s13063-021-05290-w.

## Background

It is estimated that about two-thirds of the 1.6 million people with dementia (PwD) living in Germany are cared for at home by a relative [1]. Family caregivers are essential to the quality of life of the care recipients [2]. Caring for a relative is associated with a multitude of time- and resource-intensive challenges [3–5]. Family caregivers often report that the dementia-related progressive loss of memory and physical, motor and social functions, and the manifestation of neuropsychiatric symptoms, such as agitation, depression, apathy, aggression and delusions, render caring for a person with dementia extremely stressful [6, 7].

Recent reviews confirm that family caregivers of PwD experience increased physical, psychological, emotional and social stress which, in the long term, can lead to health problems in the caregivers who are sometimes referred to as the invisible second patients [2]. Deterioration of caregivers’ health can lead to an early institutionalisation of the PwD [6, 7]. Family caregivers of people with dementia are likely to suffer from a range of health problems, such as depression, anxiety and physical illnesses [8, 9], as well as other negative conditions, such as social isolation, financial strain and poor quality of life [8, 10]. The early identification of care needs and the associated initiation of care services (e.g., visits to the doctor, joining self-help-groups, physiotherapy, or vacation replacements) can ameliorate perceived stress and prevent the occurrence of health problems among family caregivers [10, 11].

According to Murray, a need refers to a condition or experience of a problem combined with the desire to change it [12]. Based on this definition, unmet care needs of family caregivers arise from (a) the perception of a problem and (b) the simultaneous realisation that this problem is not addressed adequately by healthcare services, resources, or support services. Recent systematic reviews of qualitative and quantitative studies examining unmet care needs show that family caregivers of PwD frequently report unmet needs regarding their insufficient knowledge about dementia, available services, social, legal and financial matters, social integration and their own physical and mental health [13, 14].

Cross-sectional data from a general practitioner (GP)-based, cluster randomised, controlled intervention study conducted by our group [15] showed that 76% of family caregivers had at least one unmet care need in one of the abovementioned areas. Furthermore, the care needs of family caregivers arose from two sources: either from the care responsibilities for the PwD or from the personal needs of the family caregiver [16]. To ensure that family caregivers receive the support and care they need, regardless of where they live, their educational and socioeconomic status, problems need to be identified early and managed efficiently. Time constraints, skilled labour shortage, lack of information transfer between healthcare professionals regarding available services and lack of services in rural areas render the use of digital information and support systems essential to identify unmet needs of caregivers and appropriately address each of those in an individualised care management plan. Practitioners in general medicine have a key role in this process, because they are usually the primary healthcare contact for family caregivers. GPs often know the family caregivers for many years and can, therefore, identify changes in strain and stress as well as imminent care needs. This requires, however, that healthcare professionals not only recognise and identify unmet care needs of family caregivers of PwD but also inform them about appropriate regional care offers and provide advice on how they can access and receive them. For this, effective identification of caregiver needs and communication and collaboration between healthcare professionals need to be optimised digitally. The need for digital innovations in healthcare has been further amplified by the present COVID-19 pandemic which renders the provision of dementia services difficult if not impossible to maintain in the current healthcare structures. Given that healthcare services are often scarce, not known or not available in rural areas, the German federal state of Mecklenburg-Western Pomerania (MV) qualifies for testing the effectiveness of a digitally supported care management programme to improve healthcare services for family caregivers of people with dementia.

The programme will be supported by a tablet PC-based care management system (CMS) which will aid in generating and conducting an individualised care management plan for family caregivers. First, the CMS will identify unmet needs of family caregivers of PwD on the basis of a standardised self-administered assessment and then generate an individualised care plan based on predefined algorithms triggered by the caregivers’ answers. Qualified study nurses in collaboration with the treating GPs will then carry out the individual care plan as part of the care management programme.

This trial has been designed to assess the clinical use and cost-effectiveness of a digitally supported care management programme, based on recent research suggestions [16], own research [17–20], clinical guidelines [21] and current clinical practice in Germany.

## Methods

### Trial objectives

The primary objective is to examine the effectiveness of a digitally supported care management programme to reduce unmet needs and improve quality of life in caregivers for PwD between a group receiving the intervention and a wait-list group receiving usual care. The secondary objectives are to assess cost-effectiveness of the programme and to examine whether the care management programme is associated with improvements in participants’ perceived burden, social support and use of services and resources.

### Study design and sites

This trial is a multisite, longitudinal, cluster randomised, controlled interventional trial (cRCT) with two data assessment points (baseline assessment and six months follow-up) in a wait-list control group design. The intervention will be initiated in GP offices, specialist practices and memory clinics across the German federal state of Mecklenburg-Western Pomerania.

Throughout the study, an advisory board will convene at least annually. The advisory board will oversee the conduct of the study and provide feedback. Family caregivers of PwD, representatives of the German Alzheimer Society, GPs and specialists as well as their practice staff will be involved in the planning, development, implementation and evaluation of the study.

### Recruitment

**Fig. 1:**
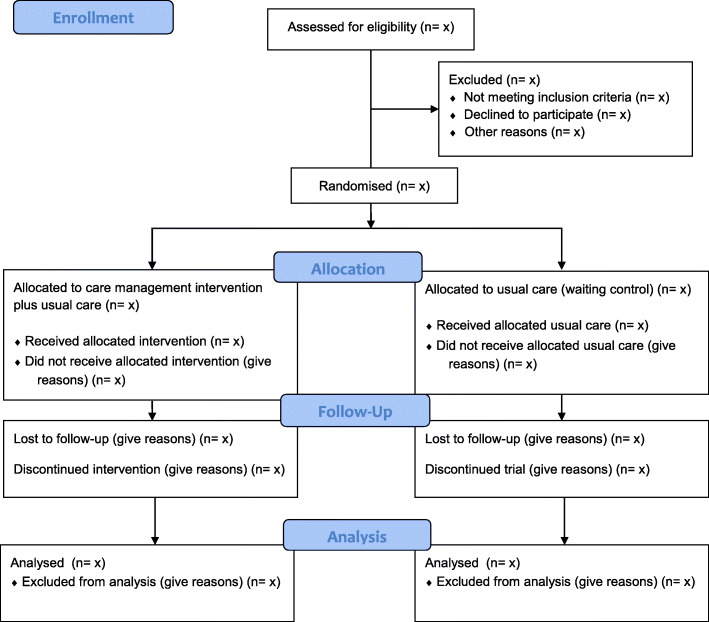
CONSORT 2010 flow diagram. The expected progress of the study

GPs and memory clinics in the federal state of MV will be contacted and invited to participate as recruitment centres. The recruitment began in October, 2020. Subsequently, caregivers will be recruited in participating GP offices and specialist practices. Participant recruitment is planned for 12 months. The expected progress of the trial is shown in Fig. 1.

### Ethical approval

Ethical approval has been obtained from the Ethical Committee of the University Medicine Greifswald (Registry number BB 120/2019) and the Ethical Committee of the University Medicine Rostock (Registry number A2020/0013).

### Study population and selection criteria

Family caregivers are eligible to participate in the study if they are above the age of 18, the caregiver of a PwD living at home, are able to speak German sufficiently to complete the baseline assessment and give written informed consent. Potential participants will be excluded if they are not living in the study region (Federal State of Mecklenburg-Western Pomerania), do not provide written informed consent, are unable to complete the self-administered baseline assessment and/or cannot be interviewed.

### Intervention

The conceptional framework of this intervention is the evidence-based Dementia Care Management as conducted and adapted in several primary care studies [17, 18, 22–24]. It consists of (i) an assessment of the health and social status of the participant at the time of recruitment, (ii) a comprehensive needs assessment at the time of recruitment, (iii) a systematic, written feedback for the participant’s treating GP, (iv) a study nurses led completion of the needs assessment at the participant’s home shortly after recruitment, (v) a collaborative consultation between the participant’s GP and the study nurse in which recommendations for treatment and care for the participant are coordinated and (vi) continuing support in reducing the participant’s unmet needs identified in the needs assessment.

The entire programme is delivered by specifically qualified study nurses in co-operation with participants’ GPs and/or specialists and will be supported by a tablet PC-based CMS. The system is a rule-based expert assessment and documentation support system that matches individual participant characteristics to recommendations for treatment and care. The system supports the identification of a participant’s unmet needs, selects corresponding participant-specific interventions and integrates these into an individualised care management plan. The CMS has been proven to support the systematic identification of unmet needs and to improve the selection of specific intervention modules [16, 25]. These will then be systematically addressed by the study nurses in co-operation with the treating GP. The CMS on its own does not replace any provisions offered by healthcare providers and is used exclusively as a tool to effectively support the individual care management programme.

### Outcomes

The primary objective is to determine the effectiveness of a digitally supported care management programme to reduce unmet needs and improve health-related quality of life in caregivers for PwD in the primary care setting.

Primary outcomes are (a) number of unmet needs and (b) health-related quality of life. They are measured as follows:

- The number of unmet needs will be assessed with a standardised assessment which is part of the tablet PC-based CMS and addresses the participants’ medical needs, home care needs, psychosocial needs and needs connected to the caregiver role. This needs assessment includes the Camberwell Assessment of Need for the Elderly (CANE) [26, 27].
- Health-related quality of life will be assessed using the EQ-5D-5L [28, 29]. This instrument comprises five dimensions, namely, mobility, self-care, usual activities, pain/discomfort and anxiety/depression. Each dimension has five levels varying from no problems to extreme problems. Each level corresponds to a 1-digit number that expresses the level selected for that dimension ranging from 1 to 5 with higher numbers indicating more severe problems. The digits for the five dimensions can be combined into a 5-digit number that describes the participant’s health status.

Secondary outcomes of this trial are the following: (a) caregiver burden, (b) social support, (c) use of medical and non-medical services and (d) the use of resources in dementia. They are measured as follows:

- Informal caregiver burden will be assessed using the seven-item version of the Zarit-Burden Interview (ZBI-7). The short version ZBI is a caregiver self-report measure to examine burden which is associated with functional/behavioural impairments in the social, psychological and physiological context and home care situation [30]. It contains seven items using a five-point scale. Response options range from 0 (never) to 4 (nearly/always). Total scores range from 0 indicating no burden to 28 indicating severe burden.
- Social support will be assessed using the Lubben Social Network Scale (LSNS-6) [31]. This scale is a self-report measure of social engagement including family and friends on a six-item scale. Total scores range from 0 to 30 with an equally weighted sum of the six items. The family and friends subscales include questions regarding the number of friends and family one has regular contact with as well as availability for help and support in private matters [32]. High scores indicate strong social networks.
- The use of medical and non-medical services includes the Questionnaire for the Use of Medical and Non-Medical Services in Old Age (Fragebogen zur Inanspruchnahme medizinischer und nicht-medizinischer Versorgungsleistungen im Alter, FIMA) [33]. The FIMA examines socioeconomic variables and other medical factors to determine health-related costs.
- The use of resources in dementia will be assessed using the Resource Utilisation in Dementia (RUD) [34].

The primary and secondary outcomes except the FIMA and RUD will be assessed at baseline (T_0_) and at the 6 months follow-up (T_6_). The FIMA and RUD will be assessed at the 6 months follow-up only (T_6_).

**Fig. 2:**
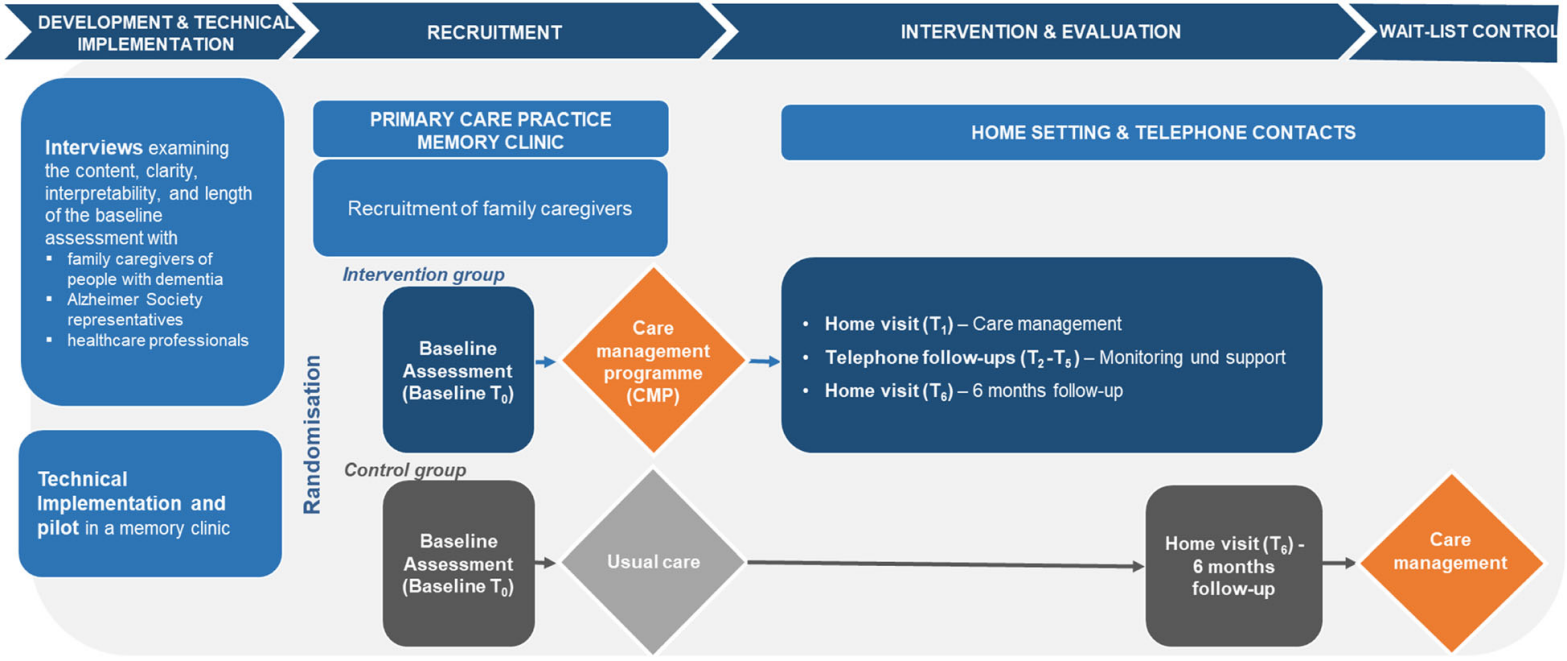
GAIN trial design

Data assessment tools for these dimensions were selected based on either their recommendation by the EU Joint Programme in Neurodegenerative Research (JPND) Working Group on Longitudinal Cohorts or common use in larger German trials such as IDemUck [35], DelpHi-MV [17, 18, 22], intersec-CM [23], or DemNet-D [36]. These instruments are validated and will allow comparability with German and international studies. An overview of the trial and assessment points of the outcome measures is provided in Fig. 2 and the SPIRIT 2013 checklist is provided in Additional file 1.

### Sample size

The estimated sample size for the study is *n*=504 participants and is based on power calculations with the NCSS programme PASS 2019 [37]. Expecting a drop-out of 30% within the study period, we assume that data of *n*=352 subjects (*n*=176 per group) will be available at the end of the study. Both groups are planned to consist of 22 clusters, each providing *n*=8 participants on average. Thus, we will be able to show a significant difference between the mean numbers of unmet needs in both groups of at least one standard deviation with a power of 80%. In this set of assumptions, the intra-class correlation is set to 0.1. The coefficient of variation of cluster sizes (COV) is 0.63, which allows variance in cluster sizes between 8 and 37. The hypothesis is tested with a one-sided test at a significance level of *α*=0.05.

### Study procedure

GP and specialist practices were selected from registries maintained by the Association of Statutory Health Insurance Physicians, with an emphasis on colleagues, who already cooperated in previous studies [17, 38]. In addition, all major memory clinics in the federal state of MV were contacted and invited to participate as recruitment centres. Participating GPs and specialists will receive a compensation fee per every participant they recruit. Participants are recruited by trained practice staff at participating practices and memory clinics during regular visits. Participants who self-identify as family caregivers of PwD and meet the inclusion criteria will be informed about the study and invited to participate in the trial by practice staff. Upon written informed consent, participants will be asked to fill out the self-administered needs assessment (baseline) on the tablet-PC while sitting in the waiting room. Informed consent materials are available from the corresponding author on request.

Consenting GPs and specialists aid in the recruitment of participants in the trial. Information about the study will be available in participating GP offices and memory clinics and interested participants will be given a participant information sheet and a consent form. Participating practices will be cluster randomised into either the intervention or control group. Participants in memory clinics will be randomised individually or will be allocated to the group of their GP practice, if that practice is already part of the trial. In both cases, the allocation to the intervention and control group will be approximately 1:1. The allocation of GP practices to intervention and wait-list control group will be computer-generated with a random number generator. Participants will then be allocated based on their GPs’ group. If the participant’s GP is not yet part of the trial, the participant will be randomised individually and the GP practice (including all following participants naming this GP) will then be allocated to this group. For participants who name a GP who is not yet randomised as part of the GAIN trial, allocation concealment will be ensured as the randomisation code will be released when the patient has been recruited into the trial, which takes place after the baseline assessment has been completed. Since this is a trial involving care management, no blinding will take place. Participating centres, participants and researchers will be aware of group allocations.

Informed consent and agreement forms will be obtained. Participating physicians will sign an agreement form and participants will sign an informed consent form. Participants will be informed that their participation is entirely voluntary and that they are free to withdraw at any time; in the event of their withdrawal, any data collected up until that point would be kept by the research team. Participants’ consent forms will be collected in paper form either by the study nurses or the respective practice staff and send to the study centre via post. As part of the consent, participants will be asked to release from confidentiality of their patient data to allow trial relevant access to this information. This includes consent allowing patient and trial relevant communication between participants’ GPs and trial staff. This trial does not involve biological specimens.

Participants who self-identify and meet the inclusion criteria will be invited to participate in the trial and upon informed consent will be given a tablet to complete the baseline assessment. The assessment uses established and valid instruments to assess participants’ medical needs, home care needs, psychosocial needs and needs connected to the caregiver role. Another part of the baseline assessment is based on the patients’ records (i.e. sociodemographic data and ICD-10 diagnoses).

After completion of the baseline assessment, the control group will receive care as usual and the participants of the intervention group will receive the intervention.

The intervention consists of an individualised systematic feedback of unmet needs ascertained in the baseline assessment issued to the treating GP. The feedback contains recommendations for treatment and care and suggests selected interventions regarding the use of care services, medication, social integration and medical treatment from a comprehensive list of intervention modules. Participants in the intervention group will be contacted immediately after recruitment by specifically qualified study nurses.

Follow-up assessments of all outcome measures will be conducted 6 months after the baseline assessment with both groups. The place and time of the assessments will be chosen based on the highest possible convenience of the participants. The progress and documentation of the intervention will be monitored by the study coordination. If required, the study coordination and the PI will make the final decision to terminate the trial. The trial is reported in accordance with the recommended Standard Protocol Items for Interventional Trials [39] and is registered at ClinicalTrials.gov (NCT04037501).

#### Intervention group

The recruitment and baseline assessment will take place in the primary care setting, at memory clinics and practices. The intervention will be administered face-to-face during visits in participants’ homes (at 2 weeks and 6 months post-randomisation, T1 and T6) and by telephone (monthly over a period of four months, T2-T5) (see Fig. 2).

The intervention group will receive a digitally supported care management programme. The basis of this programme is the tablet PC-based CMS. Upon the GP’s judgement, some or all of the CMS-recommendations and possibly further intervention recommendations will be included in the individualised care plan. Both, the assessment results and the GP’s care plan will be used by the study nurses.

The study nurses will schedule appointments for a home visit within 2 weeks post-randomisation. During the home visit, the study nurses re-visit the assessment results and collect more information about the participants’ unmet needs identified, which will then be reviewed with the participants’ GPs.

The home visit is a key component of the intervention as it allows the study nurses to get an overview of the participant’s home environment and surroundings. Given their professional background and knowledge, the study nurses will be able to optimally gear the intervention to the participant and their individual situation.

In between the home visits, the management of participants’ unmet needs are monitored and actively supported by four monthly telephone contacts. In these contacts, the progress of addressing and eliminating the unmet needs identified is discussed and is recorded in the CMS. The study nurses will also assess and record adherence as well as barriers and supporting factors for successfully alleviating caregivers’ unmet needs.

#### Wait-list control group

After the baseline assessment, the wait-list control group will receive usual care for 6 months. At the 6 months follow-up assessment, participants in the wait-list control group will receive their assessment results and intervention recommendations. Participants in the control group will have access to their usual care. In the standard German care pathway, family caregivers, as any patient, may receive general advice and/or treatment from their GPs or specialists if they actively seek it. Regarding their caregiver roles, they can access online information and self-help groups offered by the Alzheimer Society and other health organisations and charities. However, usual care does not normally include any specific or individualised care management programme as evaluated in this trial.

#### Data collection

Data will be collected in GP practices and memory clinics via a tablet PC-based, self-administered baseline assessment as well as by extraction from patients’ records. Six months after the intervention, tablet PC-based structured interviews will be conducted in participants’ homes. All assessments will be standardised using validated and reliable questionnaires. Data will be end to end encrypted and transferred to a central study database.

To record intervention recommendations provided, a success monitoring system enables standardised documentation of the intervention and the process and success of recommendations including their facilitators and barriers.

The study site will make every reasonable effort to retain participants for the entire study period of 6 months. Home visits and telephone contacts will be scheduled at times convenient for the participant. It is estimated that the drop-out rate will be at 30%. Participants are informed that all data collected up until the withdrawal of consent will be kept and used.

#### Quality assurance and safety

A scientific advisory board was installed to ensure a high quality of this trial. This board consists of experts and representatives in the field who will be meeting twice during the trial. The first meeting was held in February 2020 to discuss the design and the implementation of the trial. A second meeting will be held in February 2021 to discuss the first results and to receive feedback and advice on the scientific analysis.

Regular supervision will be provided to the study staff to ensure adherence to the study protocol, to maintain the quality of intervention delivery and to improve methods, knowledge, practice and skills. Using the tablet PC-based CMS will support a degree of standardisation of the intervention.

The data management of this trial is in accordance with the current version of the Data Protection Concept of the Institute for Community Medicine in Greifswald. This concept is approved by the State of Mecklenburg-Western Pomerania’s data safety and freedom of information office. The Data Protection Concept of the Institute for Community Medicine contains specific regulations regarding cooperation with the German Center for Neurodegenerative Diseases (DZNE) at Rostock/Greifswald and other partners.

Demographic and assessment data will be collected in GP and specialist practices as part of a digitally supported system. Only researchers involved in the trial will have access to the trial data. After the completion of the trial, personal data will be deleted and only the anonymised assessment data will be kept. This data will be kept in a password secured place and will only be accessible to the researchers.

The Data Monitoring Committee (DMC) will be a group which has no other involvement with the intervention. Members of this committee will include researchers and healthcare professionals. The committee will safeguard the interests of trial participants and monitor the overall progress, validity, credibility and conduct of the trial.

#### Publication and dissemination policy

The presentation and reporting of the trial will be in accordance with CONSORT guidelines [40]. Dissemination of the research findings will aim to cover as many channels as possible to ensure that participants, carers, healthcare professionals, researchers and the public are informed. Caregiver of people with dementia will be informed via the Alzheimer’s Association and newsletters. Findings will be published in peer-reviewed scientific journals and disseminated at national and international conferences such as the Alzheimer’s Association International Conference (AAIC) and Clinical Trials on Alzheimer’s Disease (CTAD).

### Statistical analysis

The primary between-group comparisons will be based on analysing participants as initially randomised without imputation of missing data. A full analysis plan will be developed prior to completion of data collection and discussed and consented among all research partners.

Descriptive statistics of demographic and clinical data at baseline will be used to examine differences between both groups. The primary statistical analysis will be intention-to-treat (ITT) including all individuals providing baseline and follow-up values of the outcome variables. ‘As randomised’ analyses will be performed, considering outcome data obtained from all participants regardless of protocol adherence.

To investigate whether drop-out after the baseline assessment may be systematic and influences the results, we will run multivariable logistic regressions with drop-out (yes/no) as dichotomous outcome. The study group, sociodemographic variables, and clinical parameters based on the screening will be included as predictors. These analyses will be performed three times for (1) drop-out overall, (2) drop-out due to death and (3) drop-out due to withdrawal of informed consent.

To describe the study sample, appropriate summary statistics such as the mean, standard deviation, median, minimum and maximum for continuous variables and frequencies and percentages for categorical data will be used. The primary analyses will be conducted by using separate generalised linear models to test intervention effectiveness. The main outcome variables at the primary follow-up (6 months post-randomisation) will be the dependent variables. The model specification will correspond to the scale level of the outcome variable under investigation: number of unmet needs and health-related quality of life. The models will be adjusted for age, sex and living situation of the participants. The study group is the predictor of interest (usual care vs. intervention). The baseline value of the primary outcomes will be included as a covariate to reduce residual variance and to account for inter-individual variance at baseline. A positive intervention effect is defined as a significant regression coefficient (one-sided test) of the study group variable. To improve the quality of the regression models, possible interaction effects will be analysed for study group, age group, living situation and clinical parameters as sensitivity analyses.

#### Health economic evaluation

Economic evaluations will be conducted to determine the cost-effectiveness of the care management programme compared to usual care. The healthcare resource utilisation and corresponding unit costs are used to calculate the costs from a public payer perspective using the FIMA questionnaire [33], as well as the societal perspective that includes informal care and caregiver productivity losses using the RUD questionnaire [41]. Preference-based health-related quality of life will be measured using the EQ-5D-5L [28] to estimate quality-adjusted life years [42, 43]. The incremental cost-effectiveness ratio (ICER) will be calculated using the incremental cost per (i) one unit caregiver burden saved, (ii) one unit quality of life gained and (iii) quality adjusted life years (QALY) gained by the care management programme compared with usual care. We will calculate the probability of the care management programme being cost-effective at a wide range of willingness-to-pay (WTP) margins (for example 0€ to 160,000€ per QALY gained) [44, 45]. The main results will be displayed using a cost-effectiveness plane and a cost-effectiveness acceptability curve [44]. Different sensitivity analyses will be conducted to reflect the degree of uncertainty in the ICER estimates. Differences in the cost-effectiveness due to socio-demographic and clinical differences will be assessed within a subgroup cost-effectiveness analysis. The methods used for this analysis will be consistent with those of published methodological guidelines for undertaking economic evaluations [46].

## Discussion

The GAIN study is of high relevance for family caregivers of PwD. It addresses frequently inadequate treatment and care for this population, especially for those living in rural areas. Caregivers are often not aware of available information, support and regional offers or services they can use. Similarly, healthcare professionals may not always have an overview of regional offers and services. Implementing digital support systems in healthcare processes has never been more imperative and essential to adapt to the forthcoming demographic changes. This priority has been intensified by the present pandemic which rendered the provision of dementia services difficult if not impossible in the current healthcare structures.

The care management programme developed and tested in this trial may lead to improvements in caregivers’ health, health-related quality of life, caregiver burden, social support and the use of medical and non-medical services. Making use of evidence-based methods, it is assumed that the intervention of this trial will improve treatment and care of family caregivers of PwD in primary care.

The trial will allow in-depth analyses of mediating and moderating effects for different health outcomes of family caregivers of PwD. Moreover, by identifying and addressing health risk factors, we will provide evidence that can be used to improve future interventions and, ultimately, healthcare for this important group.

The study will provide insights into the kind and number of unmet needs of family caregivers of PwD and the facilitators and barriers to reduce these needs. This knowledge might influence concepts on how to systematically support informal caregivers in the care of their relatives with dementia. Evidence regarding the usefulness and effectiveness of a tablet PC-based care management programme will be provided, and if effective, this has the potential to be implemented in routine primary care to improve the overall quality of life of family caregivers of people with dementia.

Finally, the results of this trial will not only be relevant for family caregivers of PwD but important aspects can likely be extended or adapted to improve the experience of family caregiving in general.

### Trial status

The trial is at the stage of recruitment; first participant-in: October 2020. Participant recruitment is planned for 12 months, until October 2021. Protocol Version 12.0, 12 April 2021.

## Supplementary Information

**Additional file 1.** SPIRIT 2013 checklist.

## Data Availability

There is no plan to provide public access to the data collected as part of this trial. Researchers who request the full protocol and/or the dataset may be granted access to anonymised data. Please contact the corresponding author with a reasonable request.

## Abbreviations

CMS: Care management system
cRCT: Cluster randomised controlled trial
DelpHi-MV: Dementia: life- and person-centered help in Mecklenburg-Western Pomerania
DMC: Data Monitoring Committee
EQ-5D-5L: EuroQol-5 Dimension
FIMA: Questionnaire for the Use of Medical and Non-Medical Services in Old Age
GP: General Practitioner/General Physician
ICER: Incremental cost-effectiveness ratio
ITT: Intention-to-treat
JPND: EU Joint Programme in Neurodegenerative Research
LSNS: Lubben Social Network Scale
PwD: People with dementia
QALY: Quality-adjusted life years
RUD: Resource Utilisation in Dementia
ZBI: Zarit-Burden Inventory
