# Correction to: Effectiveness of a digitally supported care management programme to reduce unmet needs of family caregivers of people with dementia: study protocol for a cluster randomised controlled trial (GAIN)

**DOI:** 10.1186/s13063-021-05395-2

**Published:** 2021-07-06

**Authors:** Olga A. Klein, Melanie Boekholt, Dilshad Afrin, Christina Dornquast, Adina Dreier-Wolfgramm, Armin Keller, Bernhard Michalowsky, Ina Zwingmann, Stefan Teipel, Jochen René Thyrian, Ingo Kilimann, Wolfgang Hoffmann

**Affiliations:** 1 German Center for Neurodegenerative Diseases (DZNE), site Rostock/Greifswald, Rostock, Germany; 2 German Center for Neurodegenerative Diseases (DZNE), site Rostock/Greifswald, Greifswald, Germany; 3 Department for Psychosomatic and Psychotherapeutical Medicine, University Hospital Rostock, Rostock, Germany; 4 European University of Applied Sciences (EU FH), Rostock, Germany; 5 Hamburg University of Applied Sciences (HAW), Faculty of Business and Social Sciences, Department of Nursing and Management, Hamburg, Germany; 6 Institute of Medical Psychology and Medical Sociology, Medical Faculty, University of Rostock, Rostock, Germany; 7 Institute for Community Medicine, Section Epidemiology and Community Health, University Medicine Greifswald, Greifswald, Germany

**Correction to: Trials 22, 401 (2021)**

**https://doi.org/10.1186/s13063-021-05290-w**

Following the publication of the original article [1], we were notified that the affiliations for some of the authors needed amendment.

The original article has been corrected.
