# Supplementary material for: Effectiveness of a digitally supported care management programme to reduce unmet needs of family caregivers of people with dementia: study protocol for a cluster randomised controlled trial (GAIN)
Source: Trials. 2021 Jun 16;22:401. doi: 10.1186/s13063-021-05290-w (PMC8206900; doi:10.1186/s13063-021-05290-w)
Supplement: Supplementary file 1 — Additional file 1. SPIRIT 2013 checklist. [file 13063_2021_5290_MOESM1_ESM.doc]

| **GAIN STUDY PERIOD** | | | | | | | | | | | |
| --- | --- | --- | --- | --- | --- | --- | --- | --- | --- | --- | --- |
|  | **Enrolment** | **Allocation** | | **Baseline** | **Post-allocation** | | | | | **Close-out** | **Wait-list group** |
|  |  | **Pre consent** | **Post consent** |  |  | | | | |  |  |
| **TIMEPOINT**** | ***t_0_*** | ***t_0_*** | | ***t_0_*** | ***t_1_***  ***_home visit_*** | ***t_2_***  ***_telephone_*** | ***t_3_***  ***_telephone_*** | ***t_4_***  ***_telephone_*** | ***t_5_***  ***_telephone_*** | ***t_6_***  ***_home visit_*** | ***t_6_***  ***_home visit_*** |
| **ENROLMENT:** |  |  |  |  |  |  |  |  |  |  |  |
| **Eligibility check** | X |  |  |  |  |  |  |  |  |  |  |
| **Informed consent** | X |  |  |  |  |  |  |  |  |  |  |
| **Recruited at GP practice** | X | X |  |  |  |  |  |  |  |  |  |
| **Recruited at specialist/memory clinic** | X |  | X |  |  |  |  |  |  |  |  |
| **INTERVENTIONS:** |  |  | |  |  |  |  |  |  |  |  |
| **Intervention Group** |  |  | |  |  |  |  |  |  |  |  |
| **Control Group: care as usual/ wait-list group** |  |  | |  |  |  |  |  |  |  |  |
| **ASSESSMENTS:** |  |  | |  |  |  |  |  |  |  |  |
| **Socio economic data** |  |  | | X |  |  |  |  |  |  |  |
| **Health-care related data** |  |  | | X |  |  |  |  |  | X | X |
| **CANE, EQ-5D-5L, ZBI-7, LSNS-6** |  |  | | X |  |  |  |  |  | X | X |
| **FIMA, RUD** |  |  | |  |  |  |  |  |  | X | X |
| **Documentation of intervention progress – only intervention group** |  |  | |  | X | X | X | X | X | X |  |
